# ASSISTING DEMENTIA FAMILY CAREGIVERS WITH INTERACTIVE AI TECHNOLOGY

**DOI:** 10.1093/geroni/igae098.0915

**Published:** 2024-12-31

**Authors:** Lin Jiang, Fei Sun, Xiayu Chen, Yali Feng

**Affiliations:** University of Texas-Rio Grande Valley, Edinburg, Texas, United States; Michigan State University, Haslett, Michigan, United States; University of Illinois Urbana-Champaign, Champaign, Illinois, United States; University of Illinois Urbana-Champaign, Champaign, Illinois, United States

## Abstract

The escalating prevalence of Alzheimer’s and related dementias (ADRD) presents a global health crisis, straining family caregivers. This study, guided by the Unified Theory of Acceptance and Use of Technology (UTAUT), examines the role of artificial intelligence (AI) in assisting ADRD family caregivers through a mixed-methods approach. It involves qualitative analysis of marginalized dementia caregivers’ experiences in the technology era and a systematic review of interactive AI evidence in dementia care. Qualitative insights from six focus groups and 24 individual interviews highlight the need for adaptable AI solutions, particularly for emotional and social support. Systematic review results underscore various AI applications, their acceptance, benefits, and challenges, including technological literacy, trust issues, limited access, and cultural considerations. The discussion emphasizes interdisciplinary collaboration and caregiver involvement in AI development. The study concludes by emphasizing AI’s transformative potential in dementia care, advocating for collaborative research efforts and innovative services to support diverse caregiver groups and their ADRD care recipients.

